# Association between birth order and birthweight percentile: population-level and within-mother analyses in a large birth cohort

**DOI:** 10.1007/s00404-026-08551-8

**Published:** 2026-08-13

**Authors:** Shanny Kolp-Asis, Oshrit Shtossel, Roi Yozevitch, Karin Edut, Ariel Lasry, Avinoam Tzabari, Nissim Arbib

**Affiliations:** 1 Maternal-Fetal Medicine Unit, Department of Obstetrics and Gynecology, Mayanei Hayeshua Medical Center, Bnei Barak, Israel; 2 Gray Faculty of Medical & Health Sciences, Tel Aviv University, Tel Aviv, Israel; 3 Department of Mathematics, Bar-Ilan University, Ramat Gan, Israel; 4 Department of Computer and Software Engineering, Ariel University, Ariel, Israel; 5 Department of Obstetrics and Gynecology, Meir Medical Center, Kfar Saba, Israel; 6 Department of Computer Science, College of Management, Rishon LeZion, Israel

**Keywords:** Birthweight percentile, Parity, Birth order, Fetal growth, Pregnancy, Cohort study

## Abstract

**Background:**

Parity is associated with differences in birthweight, but the magnitude and consistency of this association when expressed as birthweight percentiles remain incompletely characterized.

**Objective:**

To evaluate the association between birth order and birthweight percentile at both the population-level and within mothers across successive pregnancies.

**Methods:**

This retrospective cohort study included 130,718 singleton live births from 55,057 women delivering at a single tertiary medical center between 2012 and 2024. Birthweight percentiles were calculated using a national sex-specific reference. Linear mixed-effects models with a random intercept for mother were used to evaluate the association between birth order and birthweight percentile, adjusting for maternal age, infant sex, and maternal diabetes. Complementary longitudinal within-mother analyses evaluated changes in birthweight percentile across successive pregnancies.

**Results:**

Higher birth order was associated with higher birthweight percentile (*β* ≈ 0.074 SD per birth order increment, *p* < 0.001), corresponding to an average increase of approximately 3 percentile points per birth. The largest increase occurred between the first and second births, followed by progressively smaller but persistent increases with advancing birth order. This pattern was consistent across subgroup analyses and complementary within-mother analyses.

**Conclusions:**

Higher birth order was consistently associated with higher birthweight percentile both across the population and within mothers. The association followed a distinct nonlinear pattern, with the largest increase occurring between the first and second pregnancies and progressively smaller but persistent increases thereafter. These findings provide a more nuanced understanding of parity-associated variation in fetal growth across successive pregnancies.

**Supplementary Information:**

The online version contains supplementary material available at https://doi.org/10.1007/s00404-026-08551-8.

## What does this study add to the clinical work

**Table:** 

By comparing successive pregnancies within the same woman, this study demonstrates that birthweight percentile increases in a nonlinear pattern, with the largest increase occurring between the first and second pregnancies followed by smaller but persistent increases thereafter. This provides a more refined framework for interpreting fetal growth across successive pregnancies.	

## Introduction

The association between parity and birthweight has been extensively studied, with robust evidence indicating that infants born to nulliparous women have lower birthweights and birthweight percentiles compared to those born to multiparous women [1–5]. This relationship persists after adjustment for maternal sociodemographic and clinical factors [6, 7].

The increase in birthweight from one child to the next is greatest between a woman’s first and second pregnancy, with gains leveling off by the third birth. Large population-level studies show that first-born infants have consistently lower birthweight z-scores and percentiles, and that parity remains an independent predictor even after adjusting for sociodemographic confounders [1, 3, 7]. Within-woman longitudinal analyses further support that this increase is not solely attributable to changes in maternal characteristics, but reflects a physiological effect of parity itself [7].

The clinical relevance of parity-adjusted birthweight percentiles has been explored in studies evaluating customized growth standards. While incorporation of parity into fetal growth assessment may improve classification of small-for-gestational-age infants, its role remains debated, as adjustment for parity may reduce the proportion of primiparous women classified as SGA without necessarily improving identification of high-risk neonates [8].

Although the association between parity and birthweight has been described, the pattern of this association across successive pregnancies has not been fully characterized, particularly when evaluated using complementary population-level and within-mother approaches. We therefore aimed to characterize the association between birth order and birthweight percentile in a large cohort using a sex-specific national reference and longitudinal within-mother analyses.

## Methods

### Study design and data source

We conducted a retrospective cohort study at a university-affiliated institution, characterized by a high grand multiparity population where approximately 11,000 births occur annually between 2012 and 2024. Clinical data were extracted using Info-Assist, a structured query tool. The dataset included all singleton live births.

Inclusion criteria: all women who delivered a singleton live-born infant at our institution at ≥ 37 weeks of gestation.

### Data quality and cleaning

Implausible gestational age or birthweight identified during preprocessing were not excluded automatically. Instead, every such record was manually reviewed against the source medical record, and values were corrected accordingly prior to analysis.

Records with missing values in variables included in the analysis were excluded, resulting in a final analytic sample of *n* = 130,718.

### Outcome measure—birthweight percentile

The outcome of interest was the birthweight percentile of each newborn, calculated for its gestational age. We used the national birthweight reference to determine these percentiles, which is a sex-specific standard [9].

### Exposure—birth order

The primary exposure (predictor) was birth order of the infant, defined as the birth’s sequence number for its mother. For example, a first-born child has birth order = 1, a second-born = 2, and so on. Birth order was treated as a continuous numeric variable in analyses to estimate the effect per incremental increase (i.e. the expected change in birthweight percentile when comparing a second-born to a first-born, a third-born to a second-born, etc).

### Covariates

We adjusted for several key covariates known to influence birthweight:—Maternal age at delivery (in years, continuous), maternal diabetes, newborn sex (male or female). We included a binary indicator for maternal diabetes status (combining pregestational and gestational diabetes) during the pregnancy.

### Statistical analysis

For the primary analysis, we used Python (v3.10) and the statsmodels package (v0.14.5) to fit linear mixed-effects regression models quantifying the association between birth order and birthweight percentile. All continuous variables (birthweight percentile, maternal age, and birth order) were standardized (z-scored) prior to model fitting to facilitate comparability of coefficients across predictors. The dependent variable was birthweight percentile, and the key independent variable was birth order, with adjustment for maternal age, infant sex, and maternal diabetes. To account for correlation among births from the same mother, we included a random intercept for maternal identity, referred to as “Group variable”. Although birth order and maternal age could be expected to exhibit collinearity, diagnostic tests did not indicate problematic multicollinearity, as assessed using Spearman rank correlation, variance inflation factors (VIF) and sensitivity tests with and without maternal age.

We fitted the mixed model on three overlapping samples: (1) all births (*n* = 130,718 from 55,057 mothers), (2) births to women with at least two births (*n* = 108,842 from 33,181 mothers), and (3) births to women with at least three births (*n* = 83,970 from 20,745 mothers). Models were estimated by maximum likelihood, and effect sizes were summarized as standardized beta coefficients (*β*) with 95% confidence intervals. A two-sided *p* < 0.05 was considered statistically significant.

To evaluate whether the association between birth order and birthweight followed a linear pattern with a constant change across successive births, we performed an additional mixed-effects model in which birth order was treated as a categorical variable rather than as a continuous predictor. This model was fitted using the full cohort of births and included the same covariates and random intercept structure as the primary model. By estimating separate effects for each birth order category, this analysis allowed assessment of whether the magnitude of the association differed between successive births and whether the continuous birth-order model adequately captured the observed pattern.

### Within-mother analyses

In addition to the mixed-effects models, we performed complementary within-mother analyses to characterize how birthweight percentile changed across successive births within the same mother. These analyses were intended as descriptive assessments of longitudinal birthweight patterns and were not used as independent inferential tests.

For each mother, births were ordered chronologically according to birth date and assigned a birth-order number. We first calculated consecutive birth-to-birth transitions by comparing the z-scored birthweight percentile (or birthweight in grams, where indicated) of each birth with that of the immediately preceding birth within the same mother. For each transition (e.g., first-to-second birth, second-to-third birth), we calculated the mean change, standard deviation, and 95% confidence interval across all available mothers contributing to that transition.

To evaluate the cumulative change relative to the first birth, we additionally calculated, for each birth, the difference between the birthweight percentile of that child and the first child born to the same mother. These cumulative differences were summarized across mothers for each birth order. Analyses were performed for mothers contributing up to eight births, with results summarized according to the maximum number of births available for each comparison.

These analyses provide a within-mother visualization of the direction and magnitude of birthweight changes across successive births, complementing the population-level estimates obtained from the mixed-effects models.

As an additional exploratory analysis, we characterized individual-level within-mother trajectories. For mothers with at least three births, we calculated the Spearman rank correlation between birth order and birthweight percentile using the spearmanr function from the scipy.stats module in Python (v3.10, SciPy v1.15.3) only for women with at least 3 births. For mothers with two or more births, we additionally estimated an individual linear trend by fitting a simple linear regression of birthweight percentile on birth order using the linregress function from scipy.stats. For mothers with exactly two births, this estimate corresponds to the difference between the second and first birth. For mothers with three or more births, the slope represents the average change in percentile per additional birth order increment. All estimated correlations and slopes were summarized descriptively across mothers, including mean, standard deviation, and 95% confidence intervals, without filtering based on statistical significance.

### Ethics

This study was approved by the Institutional Review Board of Mayanei Hayeshua Medical Center (IRB approval No. 0025-21 MHMC). Informed consent was not required because of the retrospective nature of the study. All data were de-identified prior to analysis.

## Results

### Cohort characteristics

**Fig. 1:**
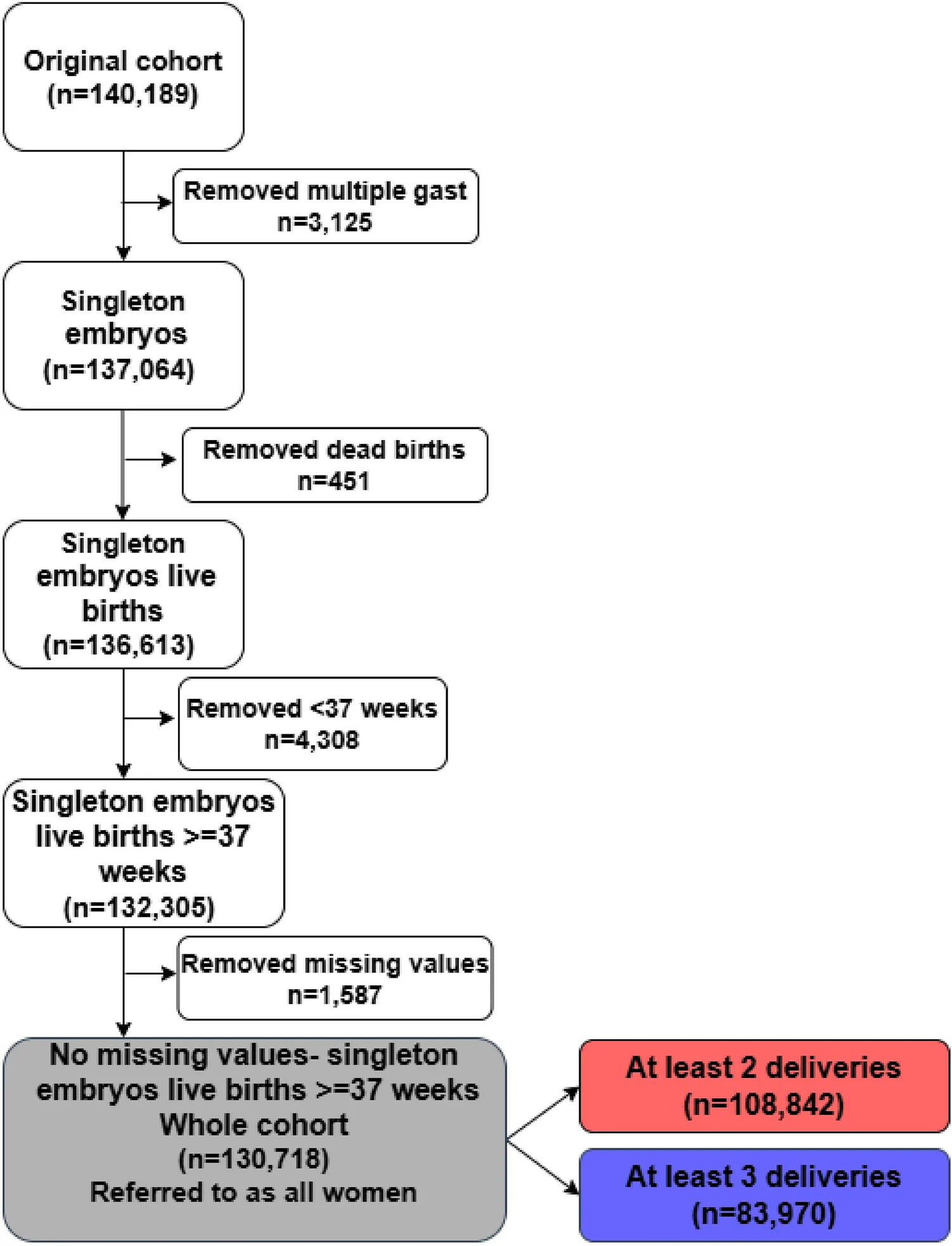
Cohort construction and filtering steps. Flowchart illustrating the derivation of the study cohorts from the original dataset (*n* = 140,189) to the all women (in gray, *n* = 130,718), the at least 2 deliveries cohort (in red, *n* = 108,842) and the at least 3 deliveries cohort (in blue, *n* = 83,970)

A total of 130,718 singleton births (after exclusions, see Fig. 1) were included, born to 55,057 mothers. As expected in a longitudinal hospital cohort, many women contributed multiple births: 60% of the mothers (*n* = 33,181) had two or more births recorded, and 38% (*n* = 20,745) had three or more. Consequently, the dataset contained a range of birth orders for analysis—42% of the births were first-born children, 25% were second-born, 16% were third-born, and the remaining ~17% were fourth or later in birth order. By design, every birth's birthweight was contextualized to its gestational age via the percentile outcome. The overall mean maternal age at delivery was 30.1 years (SD ~5.5 years). Mothers with higher-parity births tended to be older on average (mean age for first births ~28 years, vs ~32 years for third births, for example). Infant sex was almost evenly split (51.0% male infants in the cohort). Maternal diabetes (gestational or pre-gestational) was present in approximately 5.6% of the pregnancies overall; as expected, diabetes was more common in later pregnancies (for instance, 4% in first pregnancies vs. 7% in third or higher). Importantly, the majority of women in the cohort (~80%) did not have intermediate deliveries recorded at other hospitals (see Supplementary Table S1 and Figure S1).

### Birthweight percentile by birth order (unadjusted)

In unadjusted analyses, birthweight percentiles increased with birth order. The median Dolberg birthweight percentile was 49.1 for first-born infants, 55.5 for second-born infants, and 58.0 for third-born infants.

### Mixed-effects model results

**Fig. 2:**
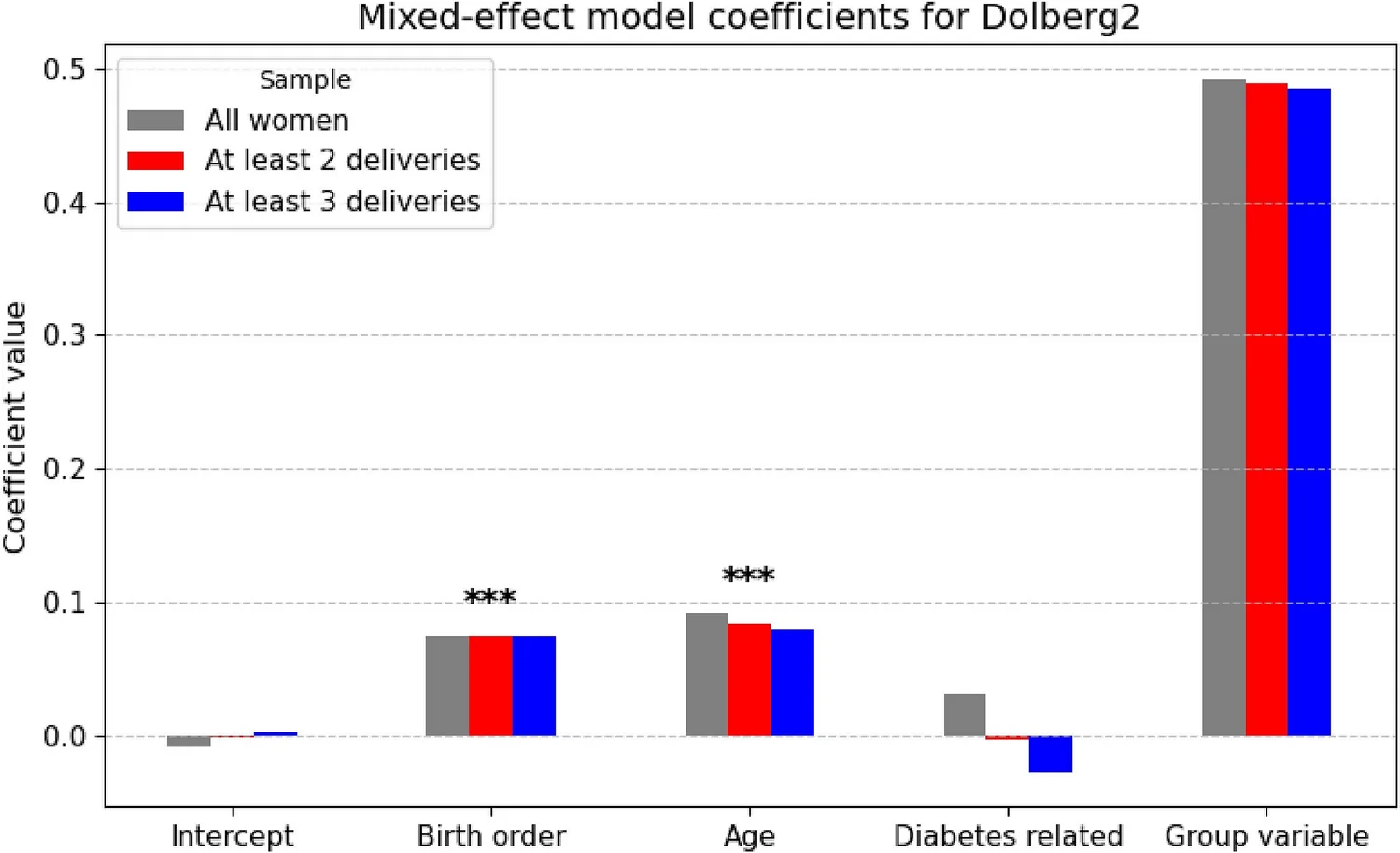
Linear mixed-effects results. Association between birth order and birthweight percentile (based on the national reference by Dollberg et al.) in the linear mixed-effects model. Each additional birth was associated with an increase of ~ 0.075 SD (≈3 percentile points) in birthweight percentile. Results are shown for the full cohort (grey), and for subgroups of women with ≥ 2 (red) and ≥ 3 births (blue), with consistent findings across analyses. Stars represent the coefficients’ *p*-values (*** *p*-value < 0.001) in all settings tested

After adjusting for covariates and accounting for within-mother clustering, birth order was significantly positively associated with birthweight percentile (Fig. 2). In the model including all 130,718 births, the *β* coefficient for birth order was approximately + 0.074 standard deviations (SD) per unit increase in birth order (*p* < 0.001). This coefficient represents the average change in birthweight percentile associated with each additional birth-order increment under a continuous birth-order specification. The mixed-effects model indicated substantial maternal-level clustering, with an intraclass correlation coefficient (ICC) of 0.498, indicating that approximately 50% of the variance in birthweight percentile was attributable to differences between mothers. To further characterize the shape of this association, we additionally fitted a mixed-effects model in which birth order was treated as a categorical variable. This analysis indicated that the relationship was not strictly linear. Compared with first births, the estimated coefficients for successive birth orders were + 0.118 SD, + 0.156 SD, + 0.183 SD, + 0.211 SD, + 0.229 SD, + 0.260 SD, and + 0.218 SD for second through eighth births, respectively (all *p* < 0.001). These estimates correspond to diminishing incremental increases after the first birth: the increase from the first to second birth was + 0.118 SD, followed by smaller increases between subsequent births (+ 0.038, + 0.027, + 0.028, + 0.018, and + 0.031 SD). The final transition (seventh to eighth birth) showed a decrease (− 0.042 SD), which may reflect increased variability at higher birth orders where fewer observations were available. Overall, the categorical model demonstrates a predominantly positive association between increasing birth order and birthweight percentile, while indicating that the magnitude of the increase is greatest between the first and second births and attenuates at higher birth orders. In practical terms, 0.0745 SD corresponds to roughly + 3 percentile points in the mid-range of the distribution (for example, an infant at the 50th percentile if first-born might be at about the 53rd percentile if born second in the family, all else being equal). Although this effect size is modest, it was highly consistent and statistically robust given the large sample size.

We observed nearly identical birth order effects in the subgroup analyses. Among women with ≥ 2 births (*n* = 108,842 births), the birth order effect was *β* ≈ 0.075 SD per birth (*p* < 0.001) after adjustment. Likewise, in the sample restricted to women with ≥ 3 births (*n* = 83,970 births), the effect was *β* ≈ 0.075 SD per birth (*p* < 0.001). The consistency of the coefficient (~ 0.074–0.075) across these analyses indicates that including or excluding mothers with only one birth did not change the estimated effect of birth order. In other words, the positive parity effect on birthweight percentile is present whether one looks at the entire population or only at families with multiple children; there was no evidence that the effect was driven solely by between-family differences.

### Covariate effects

The mixed model also provided estimates for the covariates.

Maternal diabetes was associated with a small increase in birthweight percentile in the full cohort (*β* ≈ + 0.03 SD for infants of diabetic mothers vs. nondiabetic mothers, *p* < 0.01). However, this association was not consistently observed in subgroup sensitivity analyses, where the estimated coefficients were small in magnitude and showed variation in direction, likely reflecting the limited number of diabetes-related pregnancies and reduced statistical power after restricting the cohort (Supplementary Table S2).

Maternal age showed a significant positive association with birthweight percentile after adjustment (*β* ≈ + 0.08 SD, *p* < 0.001). Importantly, in our model, the positive effect of increasing birth order was observed even while adjusting for maternal age. Because maternal age and birth order naturally increase together across pregnancies, we explicitly assessed whether this relationship could bias the model estimates (i.e., collinearity). The correlation between maternal age and birth order was moderate (Spearman *r* = 0.34, *p* < 0.001), indicating that while related, they are not redundant measures. In addition, variance inflation factor (VIF) values were low (≈1.12 for both variables), well below commonly accepted thresholds for concern, indicating that the model can reliably distinguish their separate effects.

To further ensure robustness, we performed sensitivity analyses by refitting the models both with and without maternal age. The estimated effect of birth order remained positive and statistically significant. However, the effect size was larger in models without adjustment for maternal age (see Supplementary Table S2), consistent with the positive association between maternal age, birth order, and birthweight. Therefore, excluding maternal age results in partial attribution of the age-related increase in birthweight to birth order. Since our aim was to estimate the independent association between birth order and birthweight, the model including maternal age was considered the primary analysis.

### Within-mother findings

**Fig. 3:**
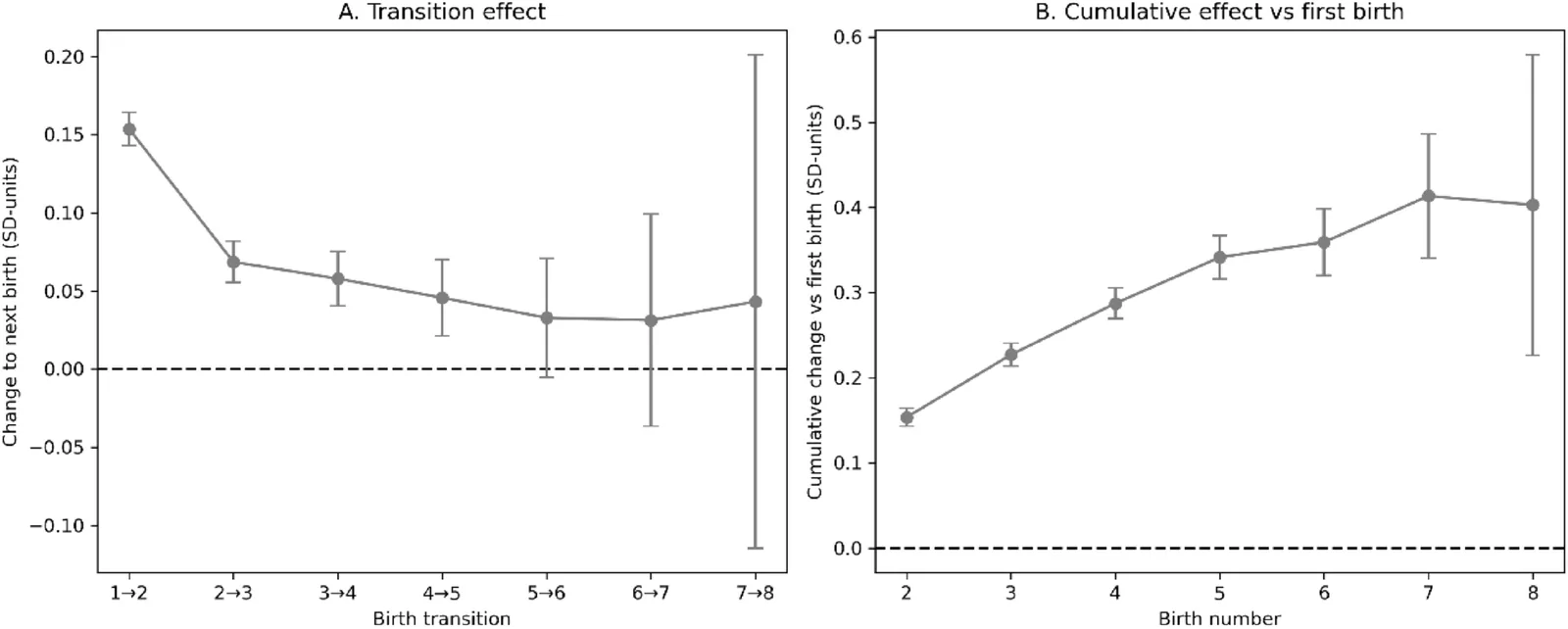
Within-mother transition and cumulative birthweight percentile changes across birth order. **A** Average within-mother transition effects, calculated as the difference in z-scored birthweight percentile (Dolberg2) between each birth and the immediately preceding birth within the same mother. Points represent the mean change for each transition, and error bars represent 95% confidence intervals. The largest increase was observed between the first and second births, followed by smaller but consistently positive increases at subsequent transitions. The wider confidence intervals at higher birth orders reflect the smaller number of mothers contributing to these comparisons. **B** Average cumulative within-mother changes in z-scored birthweight percentile relative to the first birth. Points represent the mean difference from the first-born child, and error bars represent 95% confidence intervals. Cumulative differences increased across birth orders, from approximately 0.15 SD after the second birth to approximately 0.4 SD by the eighth birth, with progressively attenuated increments at higher birth orders. For parallel results in grams, see Supplementary Figure S2

The within-mother transition analysis demonstrated a consistent but nonlinear pattern of increasing birthweight percentile across successive births (Fig. 3A and Table 1). The largest increase occurred between the first and second births, with a mean within-mother increase of approximately 0.15 SD. Subsequent birth-to-birth transitions remained positive but showed progressively smaller increments, ranging from approximately 0.07 SD between the second and third births to approximately 0.03–0.05 SD at higher birth orders.

**Table 1:** Within-mother transition and cumulative changes in birthweight percentile across birth order

Number of birth	Sample size (N)	Mean change (SD-units)	Mean change (percentile points)	Cumulative mean change first birth (SD-units)	Cumulative mean change first birth (precentile points)
2	33,181	+ 0.15	+ 4.02	+ 0.15	+ 4.02
3	20,745	+ 0.07	+ 1.79	+ 0.22	+ 5.94
4	12,093	+ 0.06	+ 1.51	+ 0.28	+ 7.52
5	6132	+ 0.05	+ 1.19	+ 0.34	+ 8.94
6	2565	+ 0.03	+ 0.86	+ 0.36	+ 9.41
7	765	+ 0.03	+ 0.82	+ 0.41	+ 10.82
8	153	+ 0.04	+ 1.13	+ 0.40	+ 10.55

The table summarizes within-mother changes in standardized birthweight percentile (SD-units) across successive births. The transition change represents the mean difference between each birth and the immediately preceding birth among mothers contributing to that comparison. Cumulative change represents the mean difference between each birth and the first-born child within the same mother. Sample size indicates the number of mothers contributing to each birth-order comparison. The results demonstrate the largest increase between the first and second births, followed by smaller but persistent increases at subsequent birth orders

The cumulative within-mother analysis showed a similar pattern (Fig. 3B and Table 1). Relative to the first-born child, later-born children had progressively higher Birthweight percentiles, with the mean difference increasing from approximately 0.15 SD for the second birth to approximately 0.40 SD by the eighth birth. However, the rate of increase attenuated after the first birth, indicating that the association between parity and birthweight percentile is not linear but follows a pattern of an initial increase followed by smaller persistent increments. Parallel analyses using birthweight in grams rather than standardized birthweight percentile showed the same overall pattern, with the largest increase occurring between the first and second births, followed by smaller subsequent increments (Supplementary Figure S2).

These findings were consistent with the categorical mixed-effects model, which demonstrated increasing birthweight percentile estimates across birth-order categories while accounting for maternal clustering and covariates. Together, the within-mother analyses indicate that the birth-order association persists within families but is characterized by diminishing returns at higher parities. As an additional exploratory assessment of individual maternal trajectories, we calculated within-mother Spearman correlations among mothers with at least three births and linear slopes among mothers with at least two births, including all mothers without filtering based on statistical significance. Among mothers with at least three births, the distribution of Spearman correlations was shifted toward positive values with an average correlation of 0.156, indicating that many mothers exhibited increasing birthweight percentiles across successive births. Similarly, the distribution of individual slopes among all mothers was centered above zero, although substantial inter-individual variability was observed (Supplementary Table S3, S4). Together, the within-mother transition, cumulative, and trajectory analyses support a parity-associated increase in birthweight percentile within families. Importantly, the pattern is not consistent with a simple linear increase across all births; rather, it suggests a strong first-to-second birth increase followed by smaller but continued increases at higher birth orders.

## Discussion

The present study demonstrates that the association between birth order and birthweight percentile is more complex than a simple linear increase across successive pregnancies. The greatest increase occurred between the first and second pregnancies, followed by progressively smaller but persistent increases with advancing birth order. Despite attenuation of the birth-to-birth increment, the cumulative difference relative to the first pregnancy continued to increase across successive pregnancies. The consistency of this pattern across multiple complementary analytical approaches strengthens confidence in the robustness of these findings. Collectively, these results provide a more nuanced characterization of parity-associated variation in fetal growth and refine current understanding of how birthweight changes across a woman's reproductive life.

Previous studies have consistently shown that first-born infants have lower birthweight than their later-born siblings and that parity is an independent determinant of fetal growth [1–10]. These findings have been observed across diverse populations and persist after adjustment for maternal demographic and clinical characteristics. More recently, Melamed et al. [2] demonstrated that the largest increase in birthweight percentile occurs between the first and second pregnancies. Our findings are consistent with this observation, as the first-to-second birth transition represented the period of greatest increase in birthweight percentile in our cohort. Importantly, by comparing successive pregnancies within the same woman, our study demonstrates that this pattern persists while minimizing the influence of stable maternal characteristics, thereby strengthening the evidence that the observed association is not solely explained by inherent differences between women. Furthermore, our study extends these observations by demonstrating that smaller positive increments remain detectable at higher birth orders, resulting in a progressive cumulative increase in birthweight percentile across successive pregnancies.

The present study extends previous knowledge by providing a more comprehensive characterization of how birthweight percentile changes across successive pregnancies. Rather than simply confirming that birth order is associated with fetal growth, our findings demonstrate that this relationship follows a distinct nonlinear pattern characterized by diminishing birth-to-birth increments but a progressively increasing cumulative effect relative to the first pregnancy. This pattern provides a more nuanced understanding of parity-related variation in fetal growth, suggesting that parity-associated differences continue beyond the second pregnancy, although their magnitude gradually decreases. The consistency of this pattern across both population-level mixed-effects models and longitudinal within-mother analyses further supports the robustness of these findings and suggests that they are unlikely to be explained solely by between-woman differences.

Although the observed effect size was modest, these findings contribute to a more refined understanding of physiological variation in fetal growth across successive pregnancies. Rather than suggesting a simple linear effect of parity, our results indicate that parity-related differences follow a dynamic pattern, with the greatest adaptation occurring after the first pregnancy and progressively smaller changes thereafter. This pattern provides additional context for interpreting fetal growth across successive pregnancies within the same woman.

Several biological mechanisms have been proposed to explain parity-associated differences in fetal growth [11–20], including uteroplacental vascular remodeling, persistent maternal cardiovascular adaptation, and changes in maternal immune tolerance across pregnancies. However, maternal metabolic changes, including postpartum weight retention and increases in prepregnancy body mass index, provide an equally plausible explanation [21–23]. Because information on maternal body mass index, gestational weight gain, postpartum weight retention, and interpregnancy weight change was unavailable in the present study, our findings cannot distinguish between these potential mechanisms. The observed association is therefore likely to reflect the combined contribution of multiple physiological and metabolic processes rather than a single underlying pathway.

Our findings should be interpreted in light of several limitations. The retrospective design precludes causal inference, and important maternal variables, including prepregnancy body mass index, gestational weight gain, interpregnancy interval, and smoking status, were unavailable. Although smoking prevalence is low in our study population, residual confounding by unmeasured time-varying maternal factors cannot be excluded. Furthermore, because this study was conducted at a single tertiary medical center with a relatively high proportion of multiparous women, the findings may not be fully generalizable to populations with different demographic characteristics or parity distributions.

The present study also has several important strengths. The large number of births, together with the substantial representation of women with higher-order pregnancies, enabled evaluation of birthweight patterns across a broad range of birth orders. More importantly, the combination of population-level mixed-effects modeling and longitudinal within-woman analyses provided complementary perspectives on the association between parity and birthweight percentile. By comparing successive pregnancies within the same woman, the study minimizes the influence of stable maternal characteristics and offers a more direct assessment of how birthweight percentile changes across reproductive life. The consistency of findings across these complementary analytical approaches strengthens confidence in the robustness of the observed pattern.

In conclusion, birth order was associated with higher birthweight percentile, but this association followed a distinct nonlinear pattern. The greatest increase occurred between the first and second pregnancies, followed by progressively smaller but persistent increases with advancing birth order. Although the incremental effect attenuated across successive pregnancies, the cumulative difference relative to the first birth continued to increase. These findings refine current understanding of the relationship between parity and fetal growth by demonstrating that parity-related differences remain detectable beyond the second pregnancy while following a pattern of diminishing incremental change. Together, these findings provide a more comprehensive description of parity-associated variation in birthweight percentile across successive pregnancies.

## Supplementary Information

Below is the link to the electronic supplementary material.

Supplementary file1 (DOCX 89 kb)

## Data Availability

The data that support the findings of this study are not publicly available due to institutional and patient privacy restrictions but are available from the corresponding author upon reasonable request and subject to institutional approval.
