# Supplementary material for: Association between birth order and birthweight percentile: population-level and within-mother analyses in a large birth cohort
Source: Arch Gynecol Obstet. 2026 Aug 13;313(1):309. doi: 10.1007/s00404-026-08551-8 (PMC13748275; doi:10.1007/s00404-026-08551-8)
Supplement: Supplementary file 1 [file 404_2026_8551_MOESM1_ESM.docx]

**Supplementary**

Table S1: Proportion of women without intermediate deliveries at other hospitals

| All women | 79.1% |
| --- | --- |
| At least 2 deliveries | 65.27% |
| At least 3 deliveries | 57.98% |

Figure S1: Comparison of estimated coefficients between the full cohort of all women (dark colors) and the restricted cohort excluding women with intermediate deliveries at other hospitals (bright colors).

Table S2: Sensitivity analysis for maternal age. Comparison of fixed-effect coefficients and variance components derived from linear mixed-effects models with and without adjustment for maternal age, evaluated in all women and in subgroups of women with at least two and at least three deliveries. Excluding maternal age increased the estimated between-mother variance component, suggesting that part of the variability associated with maternal age is captured by differences between mothers when age is not explicitly included as a covariate. The birth-order effect remained consistent in direction and magnitude across models.

| Coefficient | All women | | At least 2 deliveries | | At least 3 deliveries | |
| --- | --- | --- | --- | --- | --- | --- |
|  | With  age | Without  age | With  age | Without  age | With  age | Without  age |
| Intercept | -0.008 | -0.0001 | -0.001 | 0.003 | 0.003 | 0.005 |
| Birth order | 0.074 | 0.1137 | 0.075 | 0.1158 | 0.075 | 0.1184 |
| Diabetes related | 0.031 | 0.059 | -0.003 | 0.0154 | -0.027 | -0.013 |
| Group variable | 0.492 | 1.01 | 0.488 | 0.9935 | 0.484 | 0.9755 |

Figure S2: **Within-mother transition and cumulative birthweight percentile changes across birth order in grams.
(**A) Average within-mother transition effects, calculated as the difference in birthweight percentile (Dolberg2) between each birth and the immediately preceding birth within the same mother. Points represent the mean change for each transition, and error bars represent 95% confidence intervals. The largest increase was observed between the first and second births, followed by smaller but consistently positive increases at subsequent transitions. The wider confidence intervals at higher birth orders reflect the smaller number of mothers contributing to these comparisons.
(B) Average cumulative within-mother changes in birthweight percentile relative to the first birth. Points represent the mean difference from the first-born child, and error bars represent 95% confidence intervals. Cumulative differences increased across birth orders, from approximately 4 grams after the second birth to approximately 10.5 by the eighth birth, with progressively attenuated increments at higher birth orders

Table S3: **Distribution of within-mother slopes of standardized birthweight percentile across successive births.**
Individual maternal slopes were estimated by fitting a linear regression of z-scored birthweight percentile as a function of birth number for each mother. The table summarizes the distribution of slopes among mothers contributing at least 2 to at least 8 births, including the mean slope, standard deviation, standard error of the mean (SEM), 95% confidence interval (CI95), and number of mothers included in each analysis. No filtering based on statistical significance was applied. The mean slope was positive across all birth-count thresholds, indicating an overall within-mother increase in birthweight percentile with increasing birth order. The magnitude of the average slope gradually decreased as analyses were restricted to mothers with a higher minimum number of births, reflecting attenuation of the parity-associated increase at higher birth orders.

| Min births | Average slope (SD) | Standard deviation (SD) | Sample size (n) | SEM  (SD) | CI95  (SD)s |
| --- | --- | --- | --- | --- | --- |
| 2 | 0.114594 | 0.682736 | 33181 | 0.003748 | 0.007346 |
| 3 | 0.096093 | 0.384803 | 20745 | 0.002672 | 0.005236 |
| 4 | 0.084835 | 0.269971 | 12093 | 0.002455 | 0.004812 |
| 5 | 0.078959 | 0.206466 | 6132 | 0.002637 | 0.005168 |
| 6 | 0.070769 | 0.162876 | 2565 | 0.003216 | 0.006303 |
| 7 | 0.065776 | 0.14094 | 765 | 0.005096 | 0.009988 |
| 8 | 0.049433 | 0.124617 | 153 | 0.010075 | 0.019746 |

Table S4: **Distribution of within-mother slopes of original birthweight percentile across successive births.**
Individual maternal slopes were estimated by fitting a linear regression of birthweight percentile as a function of birth number for each mother. The table summarizes the distribution of slopes among mothers contributing at least 2 to at least 8 births, including the mean slope, standard deviation, standard error of the mean (SEM), 95% confidence interval (CI95), and number of mothers included in each analysis. No filtering based on statistical significance was applied. The mean slope was positive across all birth-count thresholds, indicating an overall within-mother increase in birthweight percentile with increasing birth order. The magnitude of the average slope gradually decreased as analyses were restricted to mothers with a higher minimum number of births, reflecting attenuation of the parity-associated increase at higher birth orders.

| Min births | Average slope (grams) | Standard deviation  (grams) | Sample size (n) | SEM  (grams) | CI95  (grams) |
| --- | --- | --- | --- | --- | --- |
| 2 | 3.000321 | 17.87548 | 33181 | 0.098133 | 0.19234 |
| 3 | 2.515915 | 10.07497 | 20745 | 0.06995 | 0.137102 |
| 4 | 2.221174 | 7.068406 | 12093 | 0.064277 | 0.125983 |
| 5 | 2.067328 | 5.405725 | 6132 | 0.069032 | 0.135303 |
| 6 | 1.852886 | 4.264439 | 2565 | 0.084201 | 0.165034 |
| 7 | 1.72216 | 3.690097 | 765 | 0.133416 | 0.261495 |
| 8 | 1.294273 | 3.262726 | 153 | 0.263776 | 0.517001 |
